# Non-medication Acquired Sticky Skin: Case Report of Idiopathic Acquired Cutaneous Adherence and Review of Medication-Induced Sticky Skin

**DOI:** 10.7759/cureus.19581

**Published:** 2021-11-14

**Authors:** Adrija K Darsha, Philip R Cohen

**Affiliations:** 1 School of Medicine, University of California San Diego, San Diego, USA; 2 Dermatology, University of California Davis Medical Center, Sacramento, USA

**Keywords:** skin, sticky, retinoid, pump, proton, medication, cutaneous, antifungal, adherence, acquired

## Abstract

Sticky skin is a dermatologic phenomenon in which the skin may cause objects to adhere to it on contact or adhere to itself or both. The entire skin can be affected in patients with sticky skin. Alternatively, just acral sites, such as the hand, can be involved. The acquisition of sticky skin has been described in patients treated with certain medications. These drugs include retinoids, proton pump inhibitors, and antifungals; they also include combination therapy utilizing an antineoplastic agent and an antifungal drug in patients with hormone-resistant prostate cancer.

The pathogenesis of acquired cutaneous adherence in patients with androgen-independent prostate cancer was postulated to be the result of therapy-induced elevation of endogenous retinoids. Retinoids have multiple biological effects on epidermal differentiation that may contribute to the pathogenesis of acquired cutaneous adherence. These include the induction of fine, granular, mucus-like deposits within and between the keratinocytes in the upper stratum spinosum and stratum corneum, modulation of lipid composition in keratinocytes, prevention of cross-linked, cornified envelope formation in keratinocytes by the inhibition of epidermal transglutaminase, and altered and decreased content of keratin within the epidermis. We describe an older man who developed non-medication acquired sticky skin (NoMasts). His acquired cutaneous adherence was considered to be idiopathic. We postulate that aging may be associated with elevated endogenous retinoid levels in older individuals and may have resulted in his sticky skin. Further investigation into these retinoid-induced effects and to what extent they promote acquired cutaneous adherence is still needed.

## Introduction

The acquisition of sticky skin has been described in patients treated with certain medications. The drugs include retinoids, proton pump inhibitors, and antifungals; they also include combination therapy utilizing an antineoplastic agent and an antifungal drug in patients with hormone-resistant prostate cancer [1-9]. In some patients with medication-induced sticky skin (MISS), an elevated level of endogenous retinoids was postulated to cause sticky skin [1].

The extent of skin involvement in patients with acquired cutaneous adherence is variable. In some patients, all of their skin can be affected. Alternatively, just acral sites, such as the hands, can be involved. The skin may cause objects to adhere to it on contact or adhere to itself or both.

An older man developed non-medication acquired sticky skin (NoMasts). His acquired cutaneous adherence was considered to be idiopathic. We postulate that aging may be associated with elevated endogenous retinoid levels in older individuals and may have resulted in his sticky skin. The features of our patient and those of individuals with MISS are reviewed.

## Case presentation

A 77-year-old man with a history of basal cell carcinoma on the nose and actinic keratoses presented for a skin check. During the visit, the patient mentioned that he had a sticky sensation to his skin. He did not have any history of hyperhidrosis and on rare occasions would take diclofenac for arthritis; he did not use any other topical or oral medications.

The sticky sensation began two years ago on his palmar hands, fingers, and antecubital fossa. Subsequently, the stickiness of these areas resolved spontaneously. Thereafter, his chest and neck became affected. He felt that his cutaneous adherence became more prominent as the duration of time between showering increased; he had showered the day prior to his appointment.

**Figure 1:**
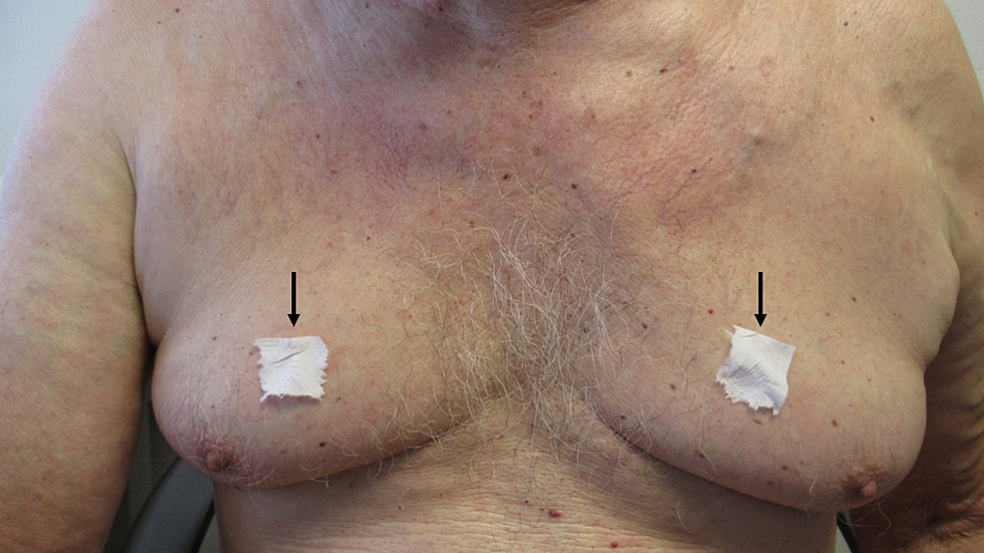
Idiopathic acquired sticky skin The chest of a 77-year-old man who developed non-medication acquired sticky skin (NoMasts). There is adherence of tissue paper that was placed on his skin as he was sitting upright.

**Figure 2:**
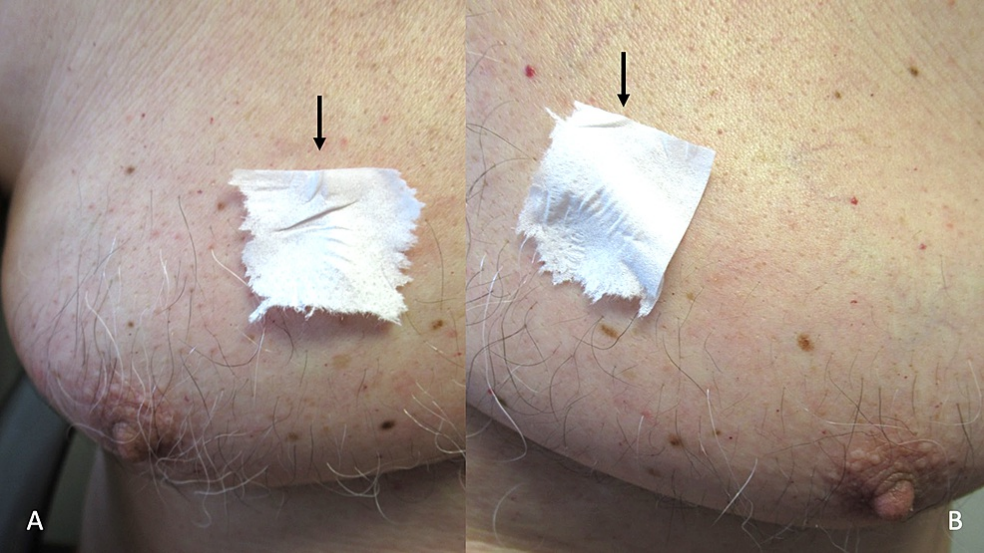
Non-medication acquired sticky skin (NoMasts) on the chest Pieces of tissue paper (black arrows) adhere to the right side of the chest (A) and the left side of the chest (B) after being placed on the skin. There is no hyperhidrosis in the affected areas.

**Figure 3:**
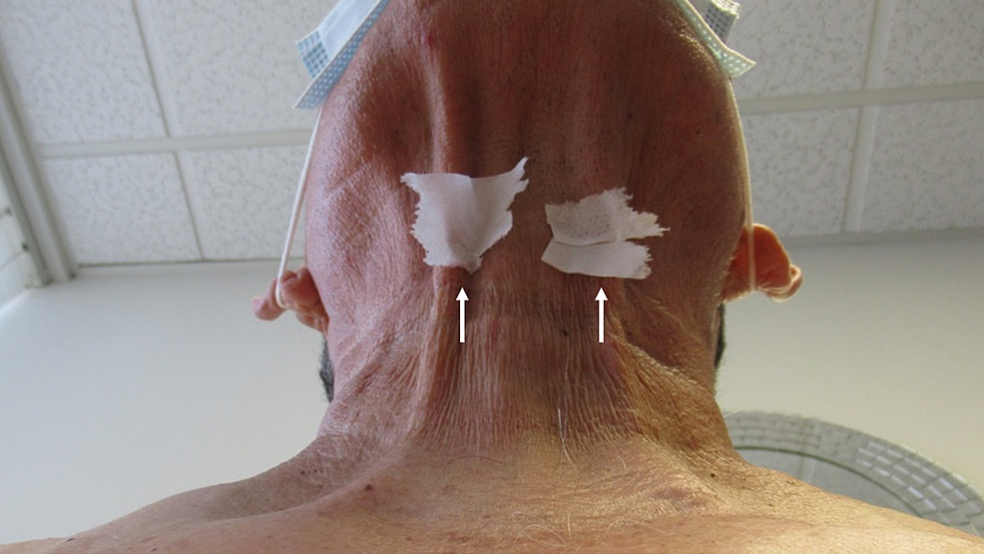
Non-medication acquired sticky skin (NoMasts) The inferior view of the neck of the patient shows adherence of pieces of tissue paper (white arrows) placed on his skin; he was sitting upright with his head positioned so that he was looking forward.

**Figure 4:**
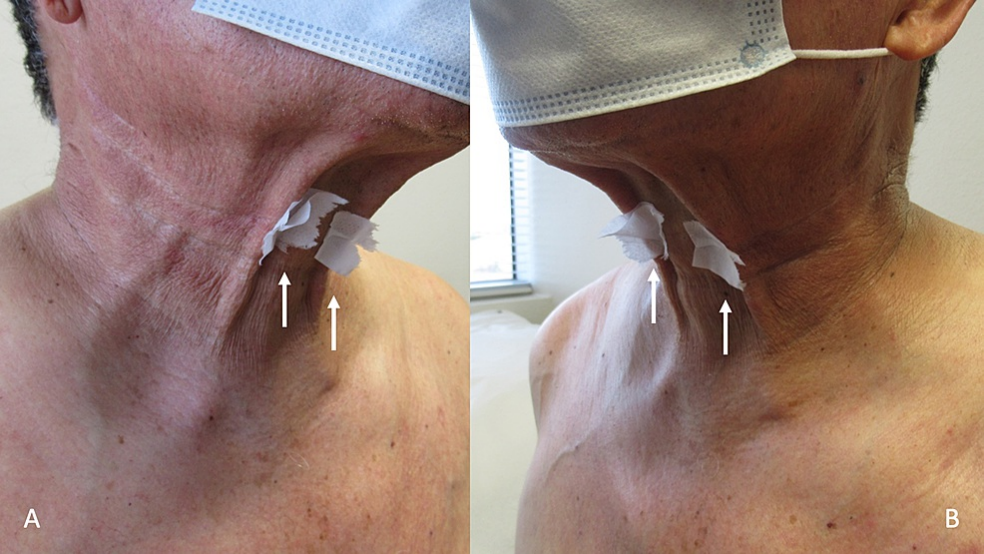
Non-medication acquired sticky skin (NoMasts) on the neck Closer views of the right side of his neck (A) and the left side of his neck (B) show adherence of pieces of tissue paper (white arrows) placed on the skin. Hyperhidrosis of the area is absent.

During his cutaneous examination, pieces of tissue paper placed on his chest (Figures 1-2) and under his neck (Figures 3-4) adhered; his skin was not moist. He declined a biopsy of the affected areas of sticky skin. Correlation of the clinical history and physical examination established the diagnosis of idiopathic, non-medication-associated, acquired cutaneous adherence.

## Discussion

Sticky skin can occur as an isolated feature or in association with hyperhidrosis. It can be exacerbated by increased ambient temperatures. Biochemical parameters such as endocrine and lipid profiles may remain within the normal range [2]. Histologic findings in sticky skin include a thickened compact stratum corneum and stratum granulosum and tall columnar basal epithelial cells that show proliferation and slight crowding [3]. A limitation of this report is that the patient did not permit biopsies of his affected sticky skin for microscopic examination.

Isolated sticky skin has previously only been reported secondary to medications (Table 1) [1-9]. However, the reported patient developed idiopathic sticky skin; we speculate that his acquisition of sticky skin may be age-related. We suggest that acquired cutaneous adherence that is not drug-related be called NoMasts. This acronym originates as follows: the “No” represents the first two letters of non; “M” represents the first letter of medication; “a” represents the first letter of acquired; “st” represents the first two letters of sticky; and “s” represents the first letter of skin.

**Table 1:** Medications associated with sticky skin

Drug	References
Antifungals	[1,6]
*Alone*	[6]
Liarozole	[6]
* Combined with antineoplastic agent*	[1]
Ketoconazole and doxorubicin	[1]
Proton pump inhibitors	[7]
Esomeprazole	[7]
Lansoprazole	[7]
Retinoids	[2-5,8,9]
*Systemic*	[2,4,5,8,9]
Acitretin	[4,8]
Arotinoid ethyl ester	[5]
Etretinate	[2,9]
*Topical*	[3]
Tretinoin	[3]

Sticky skin was first described by Ellis and Voorhees as a “subjective sensation of stickiness to the skin” in patients receiving etretinate [10]. Sticky skin has subsequently been reported in patients with psoriasis who were being treated with retinoids, patients with androgen-independent prostate cancer who were treated with ketoconazole and doxorubicin, and patients using medications listed in Table 1 [1-9].

The occurrence of sticky skin has been described in numerous patients receiving either topical or systemic retinoids. In 1993, Higgins and Pembroke described a woman with severe plaque psoriasis treated with 25 mg of etretinate daily who developed sticky skin. The patient’s whole skin was affected, but symptoms were most exaggerated on the palmar surface. Reducing the dose of etretinate to 10 mg per day produced some, but not complete, resolution of symptoms [2].

In 1999, Lockridge et al. described a 26-year-old man with acne vulgaris who developed sticky skin in areas treated with topical tretinoin. The patient had been using 1 g of oral tetracycline daily and applying tretinoin cream to his face (0.05% tretinoin) and chest (0.1% tretinoin). He had also completed a 20-week course of isotretinoin at 1 mg per kg of body weight per day and was taking daily multivitamins containing 30,000 international units of vitamin A. Physical examination revealed an ill-defined, sticky hypopigmented patch on the central chest that measured 10 cm^2^ and a sticky patch on the patient’s palm that spanned 25 cm^2^. A punch biopsy specimen from the chest showed decreased keratohyalin granules, relative acanthosis, abundant pale cytoplasm in the stratum spinosum, and crowding of the stratum basale. The sticky areas reverted to normal within 10 days of cessation of tretinoin use and multivitamin intake. Repeat biopsy revealed normalization of the epidermal changes noted previously [3].

In addition, in a study of 37 patients receiving acitretin therapy for psoriasis for 24 weeks at an average of 50 mg per day, sticky skin developed in 16% of the patients. Other typical retinoid side effects such as dry mucous membranes and peeling palms and soles were also common during acitretin treatment. Patients receiving a placebo and 10 mg of acitretin daily had fewer side effects than those taking 25 mg, 50 mg, or 75 mg of acitretin daily [4].

Sticky skin has also been reported in patients with cutaneous T-cell lymphoma. Five patients with mycosis fungoides or Sézary syndrome were given a parenteral arotinoid ethyl ester. The daily dose ranged from 0.5 to 4.0 µg per kg of body weight. The total dose received was 20 mg over a maximum of 31 weeks. The patients with mycosis fungoides had either complete (three patients) or partial (one patient) responses. However, the patient with Sézary syndrome had no response.

Treatment-associated side effects in the arotinoid ethyl ester-treated cutaneous T-cell lymphoma patients included not only systemic side effects (such as loss of appetite, bronchitis, and muscle, joint, and bone pain) but also mucocutaneous adverse events (such as cheilitis, conjunctivitis, alopecia, nail dystrophy and paronychia, dry skin and mucous membranes, and sensitive reddened palms). In addition, four of the five patients (80%) experienced increased sweating with damp sticky skin ranging in severity from one plus (a small amount in one patient) to two plus (in two patients) to three plus (strong in one patient) [5].

In 1995, Polsen et al. described sticky skin in patients with androgen-independent prostate cancer who were treated with ketoconazole and doxorubicin. The patients were treated with oral daily ketoconazole at 400 mg every eight hours and intravenous doxorubicin weekly at 20 mg/m^2^ continuously for 24 hours. Eight of the 28 patients (29%) who received treatment developed sticky skin [1].

The occurrence of sticky skin began while the patients were receiving both ketoconazole and doxorubicin. The symptoms were more severe while patients were receiving both medications and were less noticeable when they received monotherapy with ketoconazole. Four of the men had sticky skin that persisted after the discontinuation of both ketoconazole and doxorubicin. Three men had sticky skin that resolved after discontinuation of ketoconazole and doxorubicin. The wife of one of the men could not recall the duration of her husband’s sticky skin; however, his sticky thighs had only occurred for a limited duration [1].

To date, cutaneous adherence has not been described in patients who only had received either doxorubicin or ketoconazole. This observation suggests that this phenomenon of acquired sticky skin results from a combination of high-dose doxorubicin and ketoconazole [1].

Dijkman et al., in 1997, demonstrated the utility of liarozole, a derivative of imidazole and an inhibitor of the breakdown of all-trans retinoid acid, in the treatment of hormone-resistant prostate cancer patients after androgen ablation therapy. The men initially, or after four to 12 weeks, received a maximum liarozole dose of 300 mg twice daily. Adverse events included bad taste, nausea, and fatigue. In addition, all of the men experienced one or more side effects that mimicked retinoic acid toxicity; these included not only alopecia, brittle nails, dry lips, dry skin, erythema, and itch, but also sticky skin [6].

In 2016, Alkeraye et al. described two patients who developed sticky palms during the use of a proton pump inhibitor. The patients received either lansoprazole or esomeprazole. The investigators postulated that proton pump inhibitor-associated sticky palms may be a more common, albeit less reported, adverse effect from this class of medications [7].

The first patient was a man in his 30s who was being treated with 30-mg doses of lansoprazole for a gastric ulcer. In the third week of the treatment, he presented with severe palmar stickiness. The symptoms partially regressed within one month of cessation of the lansoprazole treatment [7].

The second patient was a woman in her 40s who was taking 20-mg doses of esomeprazole for gastroesophageal reflux disease. She developed sticky palms two weeks after the start of the treatment. One week after stopping the treatment, the symptoms resolved [7].

The pathogenesis of acquired cutaneous adherence remains to be established. Polsen et al. proposed that the occurrence of sticky skin in patients receiving either etretinate or ketoconazole and doxorubicin shared a common mechanism. They suggested that the activity of cytochrome P450-dependent retinoic acid hydroxylase, one of many enzymes that govern the metabolism of retinoic acid, may contribute to the etiology [1].

Ketoconazole causes antifungal activity by inhibiting fungal cytochrome P450-mediated 14a-demethylase. In-vitro and in-vivo studies in mice have shown that, at higher doses, ketoconazole can inhibit the cytochrome P450-dependent metabolism of exogenously administered retinoic acid. The endogenous plasma level of retinoic acid was also enhanced by ketoconazole inhibition of the cytochrome P450 pathway [1].

Thus, Polsen et al. postulated that the pathogenesis of acquired cutaneous adherence in patients with androgen-independent prostate cancer might be the result of therapy-induced elevation of endogenous retinoids [1]. Consistent with this hypothesis, systemic retinoids and liarozole have also been shown to increase levels of endogenous retinoids [11,12].

Retinoids have multiple biological effects on epidermal differentiation that may contribute to the pathogenesis of acquired cutaneous adherence. These include the induction of fine, granular, mucus-like deposits within and between the keratinocytes in the upper stratum spinosum and stratum corneum; modulation of lipid composition in keratinocytes; prevention of cross-linked, cornified envelope formation in keratinocytes by the inhibition of epidermal transglutaminase; and altered and decreased content of keratin within the epidermis [1]. However, further investigation into these retinoid-induced effects and to what extent they promote acquired cutaneous adherence is still needed.

In contrast to the patients with medication-associated acquired sticky skin, the current patient was taking no systemic or topical medications. Due to his advanced age, we postulate that he had developed idiopathic or age-related acquired cutaneous adherence. Consistent with Polsen et al.’s postulation, research has shown that the metabolism of endogenous retinoids changes with age. Aging research in rats show increased absorption of vitamin A by the intestines with age [13]. Additionally, older adults have been found to clear infused vitamin A half as quickly as younger adults [14]. Both phenomena can potentially lead to increased endogenous retinoids in older individuals and may contribute to the occurrence of NoMasts.

## Conclusions

Acquired cutaneous adherence can be associated with medications or idiopathy and possibly can be age-related. Medication-associated sticky skin (MISS) has been associated with antibiotics, antifungal medications, antineoplastic agents, oral and systemic retinoids, and proton pump inhibitors. MISS may be persistent or resolve within weeks to months after discontinuation of the culprit medication. Reducing the dose of the associated medication may provide some symptom relief. Non-medication acquired sticky skin (NoMasts) occurred as an idiopathic finding in our patient. It may be reasonable to postulate that he developed this acquired cutaneous adherence due to age-related changes.

## References

[1] Polsen JA, Cohen PR, Sella A (1995). Acquired cutaneous adherence in patients with androgen-independent prostate cancer receiving ketoconazole and doxorubicin: medication-induced sticky skin. J Am Acad Dermatol.

[2] Higgins EM, Pembroke AC (1993). Sticky palms--an unusual side-effect of etretinate therapy. Clin Exp Dermatol.

[3] Lockridge J, Ramos-Caro FA, Holloway K, Mullins D (1999). Tretinoin-induced sticky skin: a case report and review of the literature. Cutis.

[4] Goldfarb MT, Ellis CN, Gupta AK, Tincoff T, Hamilton TA, Voorhees JJ (1988). Acitretin improves psoriasis in a dose-dependent fashion. J Am Acad Dermatol.

[5] Mahrle G, Thiele B, Ippen H (1983). [Chemotherapy of cutaneous T-cell lymphomas with arotinoid]. Dtsch Med Wochenschr.

[6] Dijkman GA, Fernandez del Moral P, Bruynseels J, de Porre P, Denis L, Debruyne FM (1997). Liarozole (R75251) in hormone-resistant prostate cancer patients. Prostate.

[7] Alkeraye S, Baclet Y, Delaporte E (2016). Sticky palms following use of proton-pump inhibitors. JAMA Dermatol.

[8] Sarkar R, Chugh S, Garg VK (2013). Acitretin in dermatology. Indian J Dermatol Venereol Leprol.

[9] Penneys NS, Hernandez D (1991). A sticky problem with etretinate. N Engl J Med.

[10] Ellis CN, Voorhees JJ (1987). Continuing medical education (therapy): etretinate therapy. J Am Acad Dermatol.

[11] Miller WH (1998). The emerging role of retinoids and retinoic acid metabolism blocking agents in the treatment of cancer. Cancer.

[12] Bugge CJL, Rodriguez LC, Vane FM (1985). Determination of isotretinoin or etretinate and their major metabolites in human blood by reverse-phase high-performance liquid chromatography. J Pharm Biomed Anal.

[13] Hollander D, Dadulfalza V (1990). Influence of aging on vitamin A transport into the lymphatic circulation. Exp Gerontol.

[14] Krasinski SD, Cohn JS, Schaefer EJ, Russell RM (1990). Postprandial plasma retinyl ester response is greater in older subjects compared with younger subjects. Evidence for delayed plasma clearance of intestinal lipoproteins. J Clin Invest.

